# A Stochastic Model of Latently Infected Cell Reactivation and Viral Blip Generation in Treated HIV Patients

**DOI:** 10.1371/journal.pcbi.1002033

**Published:** 2011-04-28

**Authors:** Jessica M. Conway, Daniel Coombs

**Affiliations:** Department of Mathematics and Institute of Applied Mathematics, University of British Columbia, Vancouver, British Columbia, Canada; Imperial College London, United Kingdom

## Abstract

Motivated by viral persistence in HIV+ patients on long-term anti-retroviral treatment (ART), we present a stochastic model of HIV viral dynamics in the blood stream. We consider the hypothesis that the residual viremia in patients on ART can be explained principally by the activation of cells latently infected by HIV before the initiation of ART and that viral blips (clinically-observed short periods of detectable viral load) represent large deviations from the mean. We model the system as a continuous-time, multi-type branching process. Deriving equations for the probability generating function we use a novel numerical approach to extract the probability distributions for latent reservoir sizes and viral loads. We find that latent reservoir extinction-time distributions underscore the importance of considering reservoir dynamics beyond simply the half-life. We calculate blip amplitudes and frequencies by computing complete viral load probability distributions, and study the duration of viral blips via direct numerical simulation. We find that our model qualitatively reproduces short small-amplitude blips detected in clinical studies of treated HIV infection. Stochastic models of this type provide insight into treatment-outcome variability that cannot be found from deterministic models.

## Author Summary

While on successful drug treatment, routine testing does not usually detect virus in the blood of an HIV patient. However, more sensitive techniques can detect extremely low levels of virus. Occasionally, routine blood tests show “viral blips”: short periods of elevated, detectable viral load. We explore the hypothesis that residual low-level viral load can be largely explained by re-activation of cells that were infected before the initiation of treatment, and that viral blips can be viewed as occasional statistical events. To do this, we propose a mathematical model of latently-infected cells, activated cells, and virus. The model captures random fluctuations of the system as well as the mean behaviour. We estimate the time it takes for all the latently-infected cells to be eradicated. Eradication of these cells is considered a major hurdle in eliminating infection. We predict a wide range of eradication times, highlighting the importance of studying latently-infected cells. We also estimate the frequency and duration of viral blips, and find qualitative agreement with clinical studies. By refining our models, we hope to find guidelines that can be used in practise to distinguish between clinically insignificant statistical blips, and instances of drug failure.

## Introduction

HIV infection can be effectively controlled by anti-retroviral drug therapy (ART) [1], [2]. Different ART drugs inhibit different steps of HIV replication, and therefore truly effective therapy should halt viral production altogether. However, while plasma viral load is greatly decreased in patients on ART, it remains non-zero [3]–[5]. The sources of residual viremia remain under debate. One common argument is that the drugs may not be 100% effective, implying that the low-level viral load is associated with some residual viral replication. Older papers (pre-2004) present considerable evidence for this hypothesis [6]–[8]; for example, Havlir et al. [8] noted that, in patients on long-term suppressive therapy, the introduction of an improved drug into their regimen decreased the level of residual viremia.

However, the efficacy of ART drugs has improved substantially since their inception and the likelihood of substantial ongoing viral replication has correspondingly diminished. A recent phylogenetic study of virus before treatment and during structured treatment interruptions found that the viral samples were too closely related for there to have been significant ongoing replication [9]. Other studies measured residual viremia in patients on ART before and after treatment intensification, and found no change in residual viremia [10],[11] (although the latter paper intriguingly discovered signs of replication in certain patients even though their plasma viral load was maintained at extremely low levels). Together, these works indicate that there are important sources of virus in treated patients and these sources are largely independent of ongoing viral replication.

### The latent reservoir during HIV infection

There are many locations in the body from which viruses could re-emerge during drug treatment; for a review, see [6]. Here, we will focus on the important possibility that viruses may emerge from a reservoir of latently infected cells. Usually when HIV infects target cells (such as CD4+ T cells and macrophages) the result is rapid virus production and cell death. However, a fraction of infected cells are known to enter a state of latent infection [12] where virus has integrated into the host cell DNA, but there is little, if any, viral gene expression. While cells are in this state, they are unaffected by ART and viral cytopathicity, and are effectively invisible to the host immune response [13]. However, upon re-activation, latent cells begin the normal processes of viral replication and production, and become immune targets [14]. A large fraction of the latent reservoir consists of resting memory CD4+ cells [15] and therefore, reactivation could occur as part of the normal immune response to a secondary pathogen [16]. However, we do not completely understand the reasons for activation of latently infected cells and it is likely that there is a pathogen-independent component as well. Indeed, the mechanisms for latency are generally poorly understood; there are differing opinions, but no consensus to date [12], [17]–[19].

The population of latently infected cells is established as early as 10 days after symptoms of seroconversion, within a few weeks of initial infection [14]. Estimates of reservoir size differ but consistently show that latently infected cells constitute only a small fraction of the total number of T-cells [12], [20]. Unfortunately, and in spite of its small relative size, the decline of this population is slow and it is estimated that it can persist for up to 70 years [21]. This long lifetime probably arises from the intrinsic stability of resting memory CD4+ cells which is an important part of immune memory [22]. Recent evidence also indicates that latently infected cells can undergo cell division [15], potentially increasing the lifetime of the reservoir. These factors, in combination with long lifetime of the reservoir, indicate that latently infected cells are an important factor that must be addressed in the search for therapies to eradicate HIV infection [23].

### Viral blips during anti-retroviral treatment

While on successful anti-retroviral treatment (ART) for HIV, an infected individual's viral load remains non-zero [3], though it is very low and usually undetectable using standard assays that have a detection limit of 50 copies/mL in plasma. Occasionally, however, regular blood tests show viral blips: periods of detectable viral load, preceded and followed by undetectable loads. At one time there was a concern that blips might signal imminent drug failure [24], including the emergence of new, drug-resistant variants of virus [25]. However, there is a substantial body of evidence dating from the early 2000s, indicating that most blips are not associated with virological failure [26]–[28]. With that said, a recent large-scale study of 3530 Canadian patients refined these results by showing a two-fold increase in the risk of drug failure following viral blips that exceeded 500 copies/mL, but importantly, smaller blips were not associated with drug failure [29]. In this study, blips were detected at a frequency of about 0.1/patient/year. This rate is compatible with data taken from the UK during 2006–2007 [30] and is lower than the rate estimated from earlier data [31]. The reduction in blip frequency over the last decade is likely a result of improved drug efficacy.

The underlying cause of viral blips remains controversial. There is some evidence that immune activation, through secondary infection or vaccination, may be correlated with viral blips, [32], [33]. However, there have been observations of blips not associated with clinical or demographic variables. In an intensive 90-day study of 10 patients, Nettles et al. found that blips were fairly common, smaller in amplitude (mean 79 copies/mL) and short in duration (median less than 3 days), and that blip frequency was unrelated to illness, vaccination, or drug concentrations [34]. Finally, we must acknowledge that accurate detection of small-amplitude blips is bedevilled by assay variability and sensitivity [30], [34].

### Previous modeling work

There has been extensive modeling work done to characterize viral load and pathogen-immune system interaction in HIV-infected individuals. However, standard viral dynamics models do not capture residual viremia in treated patients and various modeling approaches have been applied.

Residual viremia can be captured by adding a latent cell reservoir to the standard model. Perelson et al. (1997) proposed the first model that included latent cell activation, in order to better understand decay characteristics of HIV-1-infected compartments during combination therapy [35]. This model was expanded to include a varying decay rate in the latent reservoir, and bystander proliferation in the latent reservoir, with the finding that a constant long-term activation rate for the latent reservoir, maintained through cell division, could explain residual viremia in treated patients [36]. We will use these elements in the development of our model of the latent reservoir.

Careful modelling of viral blips has been fruitful in analyzing different mechanisms of blip generation. The focus of previous models has been on blips associated with immune system activation due secondary infection or vaccination. One successful approach has been to consider short periods of sustained viral replication driven by stochastic activation of CD4+ and CD8+ T cells [37]–[39]. A further series of models including T cell expansion due to vaccination and secondary infection showed episodes of detectable viremia of long duration (2–3 months), with amplitudes in the range of several hundred copies/mL [40], [41]. Viral blips can also result from production of virus following immune activation and clonal expansion of latently infected cells [33]; latent cell activation caused by sporadic immune activation has also been modeled as a source of viral blip generation. Indeed, antigen-induced latent cell activation has been modeled and shown to be a plausible source of viral blips [42]. Most recently, Rong and Perelson (2009) proposed a model with antigen-induced asymmetric activation and division of latently infected cells [43]. Blips produced by this model are of short duration, directly related to the length of stimulation, and of variable amplitude, consistent with observations. These models produce blips of larger (100 copies/mL) amplitude, with variable durations, and frequency depending directly on user-controlled periods of immune system activation in the model. The base mechanism in these models of the production of blips is immune system activation.

### A stochastic model of latent cell reactivation and viral load during ART

As noted above, there have been observations of small-amplitude blips not associated with clinical or demographic variables [34]. Such blips can be imagined as random biological or statistical variation around a mean undetectable viral load. In order to capture this kind of stochastic effect, continuous (differential equation based) models are inadequate.

Here, we propose a continuous-time branching process model of within-host viral dynamics for a patient undergoing successful treatment. We use this formulation to derive probability distribution functions for viral load as a function of time and examine the contribution of varying latent cell activation and proliferation to viral load. Using this methodology we first consider extinction times for the latent reservoir and examine the role of limited ongoing viral replication in replenishing the reservoir. We then examine the hypothesis that stochastic activation of latently infected cells can maintain low-level plasma viremia and generate small intermittent viral blips. Finally, via Gillespie simulation of the branching process, we calculate viral blip durations.

## Methods

### Viral dynamics in treated patients

**Figure 1:**
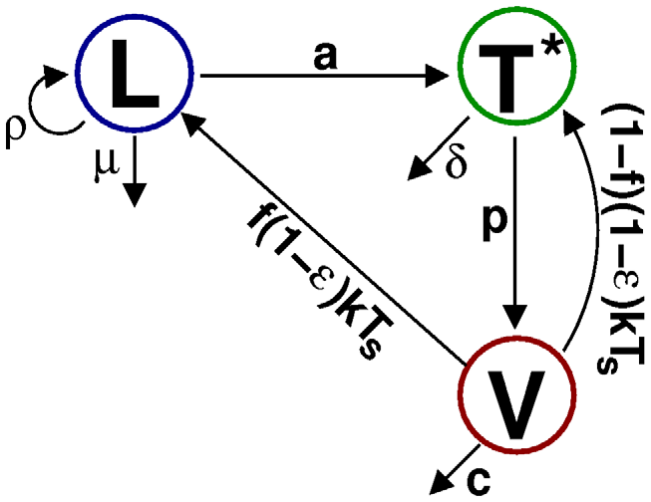
Model schematic. Latently infected cells (L) divide, die, and become activated with rates 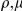 and 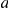 respectively. Productively infected cells (T*) die at rate 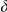 and produce virus (V) continuously, at rate 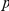. Free virions are cleared at rate 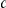 and infect healthy cells at rate 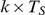, reduced by drug treatment of efficacy 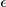. A fraction 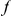 of newly infected cells become latently infected cells and the rest become productively infected cells.

We consider a simple model of latent cell reactivation, presented schematically in Figure 1. Our model has three compartments: the number of latently infected cells 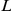, which can replicate at rate 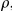 die at rate 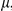 and activate at rate 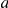 to become productively infected cells; the number of productively infected cells 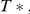 which die at rate 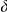; and the number of virions 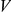, produced by productively infected cells at rate 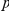, which can die at a rate 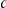. We allow for infection of new cells at rate 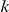, of which a fraction 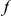 become latently infected. The efficacy of ART is given by 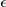. We will assume that this efficacy is very high, and that therefore the number of uninfected T-cells remains approximately constant and equal to 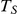. Clinical findings on viral blips show differing amplitudes [34], [44], [45]; though the small-amplitude blips were shown to be unassociated with clinical variables [34], it is possible that the larger-amplitude blips may be due to an immune response, increasing the activation rate 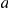 for a period of time. We will initially restrict ourselves to constant activation rate, but we consider variable 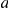 in a later section.

The mean behaviour of the system shown in Figure 1 is given by the linear system of ordinary differential equations
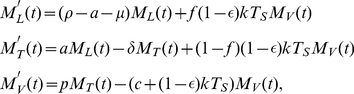
(1)where 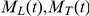, and 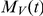 represent the mean numbers of latently infected cells, productively infected cells, and virions respectively.

### Probability distribution calculations

Our goal is to obtain probability distributions for the viral load and the size of the latent reservoir at time 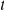. We assume that the events in the model can be described by a multi-type continuous time branching process with the rates given in Figure 1. Importantly, the model does not scale up and so any computations we perform must be over the total number of 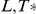 and 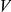 in the patient.

The transition probabilities for each process in the model are stationary in time. We therefore know that our desired probability distributions depend only on the time since the initial state, and consider
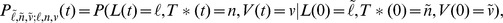
the conditional probability that there are 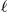 latently infected cells 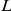 at time 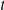, 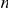 productively infected cells 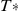 at time 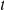, and 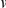 virions 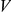 at time 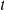 given that there were initially 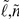, and 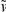 of each species respectively. Then, given a joint initial distribution on these species 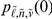, we can compute the joint probability distribution on each of these
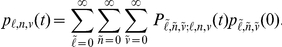
Note that, as the latent reservoir must be of finite size, 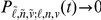 and 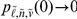 as 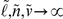.

By considering each process in Figure 1 in turn, we can derive the backwards Chapman-Kolmogorov differential equation for 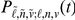 [46]:
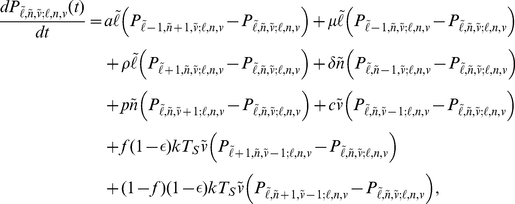
(2)with initial condition 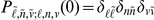 (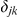 is the Kronecker delta function). Multiplying through by 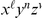 and re-indexing yields an infinite-dimensional system of nonlinear ordinary differential equations for the conditional probability generating function
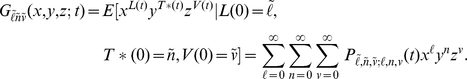
We reduce the infinite dimensional system to a system of three equations by exploiting the branching property 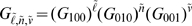 [46],
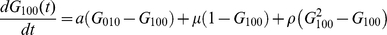


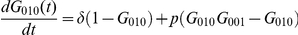


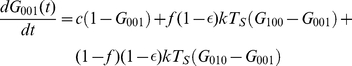
with initial conditions 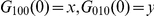 and 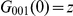. To our knowledge we cannot solve this nonlinear system analytically. Therefore to calculate 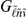, we solve numerically using a standard differential equation integrator. Once 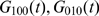 and 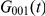 are calculated we can compute the full probability generating function, accounting for the initial distributions
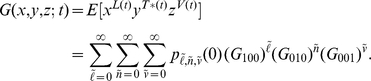
Our goal is to recover the probability distributions of latently infected cells, productively infected cells, and virions at times 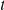. These can be recovered from the probability generating function 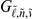 by taking derivatives. For example, the probability that there are 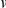 virions at time 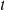 is given by
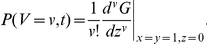
Because the distributions do not scale, we must perform computations over the total number of virions 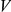. Given a mean viral load of 25 copies/ml (henceforth abbreviated as 5 c/mL) within 5L of total blood volume, we must compute 125000 derivatives to get 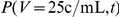
! Direct numerical differentiation would be difficult, so we exploit the Cauchy-Euler formula:
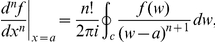
where 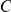 is a closed curve in complex space which contains 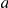, and 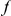 is analytic on a simply connected domain containing 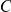. The probability generating function 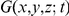 is a polynomial in 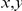 , and 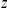 and therefore satisfies the analyticity requirement. We want to evaluate integrals at 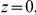 so our contour 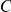 must contain the origin and it is simplest to use the unit circle, 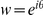, 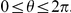 Then
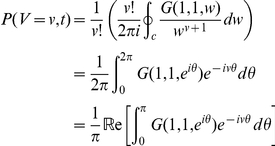
where we have used the fact that 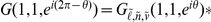, where 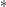 indicates complex conjugate. By this method we can calculate our probabilities via straightforward and reliable numerical integration. The same approach can be used to compute joint probability distributions.

We can also use this formulation to directly calculate cumulative probabilities. As 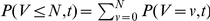, we can write
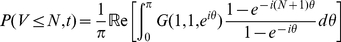
by interchanging the order of integration and summation. This final formula will be useful in calculating blip probabilities at a time 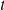, 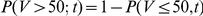.

To our knowledge this is a novel method for computing probability distributions from single- or multi-type continuous time branching processes. We thoroughly tested our method and its implementation; see Figure S1 for comparisons with Gillespie simulations.

### Extinction probabilities

We also wish to calculate the distribution of times to extinction for the latent reservoir. We choose parameters so that the probability of extinction of the latent reservoir is 1 as 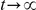. However, as clearing the latent reservoir is considered a major hurdle in clearing HIV, the distribution of times to this inevitable extinction is of interest. We obtain the cumulative probability of latent reservoir extinction directly from the probability generating function. Since 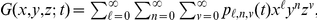


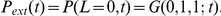
Note that the marginal probability 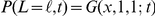. We then find the probability distribution of extinction times by differentiating,
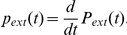
If we assume that no newly infected cells become latently infected (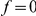) or that treatment is completely effective (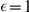), we can obtain the extinction probability analytically. In this case, the latent cell dynamics decouples from the rest of the model and can be represented as a pure birth-and-death process with master equation
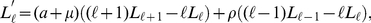
(3)where 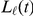 is the probability that at time 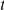 there are 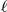 latently infected cells. This probability has the conditional probability generating function
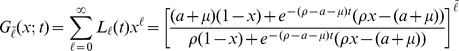
where 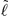 is the initial reservoir size. The cumulative distribution is 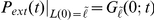 and thus we obtain
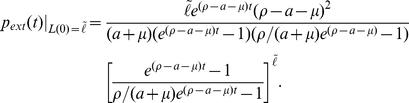
(4)Then given the initial distribution on the latent reservoir 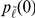 we have can compute the extinction probability 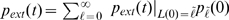. We use the analytic expression to compute latent reservoir extinction time distributions for 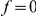 or 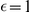. Otherwise, we work numerically.

### Parameter estimation

The parameters used for simulation results presented below are given in Table 1. In our simulations the parameters 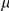, 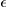 and 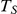 are chosen based on estimates from [36] and 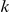 based on estimates from [47]. The decay rate of the latent reservoir is chosen so that its half-life is 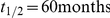, as measured in patients exhibiting viral blips [21]. For 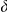, the death rate of productively infected cells, and 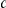 the virion clearance rate we set to estimates from [48] (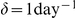) and [49] (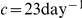), respectively. The *in vivo* viral production rate 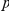 is not well established and therefore we will consider a range for this parameter. The fraction 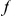 of new viral infections that result in latency is also hard to estimate, but given the small size of the latent reservoir, it is likely to be rather small. For simplicity, we choose a baseline value of 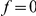. We choose the initial mean latent reservoir size 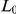 consistent with the experimental estimates [21], [50].

**Table 1:** Baseline parameter values for latent cell activation model.

Parameter	Description	Estimate
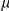	Death rate of latently infected cells	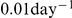
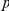	Virion production rate for a productively infected cell	5000, 10000, 20000 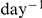
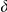	Death rate of productively infected cells	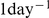
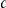	Clearance rate of free virions	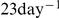
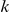	Mass-action infectivity of free virions	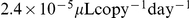
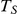	Target cells	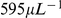
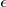	Drug efficacy	0.93
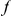	Fraction of new infections that result in latency	0
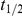	Half life of latent reservoir	60 months
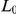	Initial number of latently infected cells	1 per 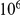 target cells

The activation rate 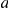 and replication rate 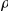 of latently infected cells remain unknown. We calculate values from the mean behaviour equations (1), taking 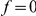. Since the dynamics of the productively infected cells and virus are more rapid than those of the latent reservoir, we can make a quasi-steady approximation to find 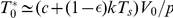. Then for an initial latent reservoir size of 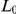, 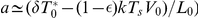. To calculate 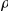, we choose the mean decay rate so that the half-life of the latent reservoir is 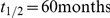. Thus we can set 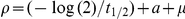. The resulting (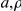) values for each production rate 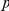 and mean viral loads of 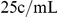 or 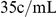 are given in Tables 2 and 3 respectively.

**Table 2:** Calculated activation and replication rates for initial mean viral load of 25 c/mL.

Production rate 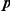	Activation rate 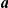	Replication rate 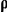
5000 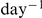	0.1513 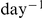	0.1609 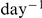
10000 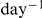	0.0546 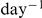	0.0643 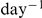
20000 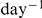	0.0063 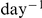	0.0159 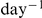

We calculate the activation rates 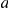 for an initial mean viral load of 25 c/mL, given the virion production rate 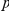. The replication rate 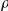 is then chosen so that the half-life of the latent reservoir is 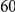 months.

**Table 3:** Calculated activation and replication rates for initial mean viral load of 35 c/mL.

Production rate 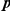	Activation rate 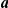	Replication rate 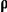
5000 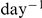	0.2118 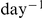	0.2214 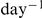
10000 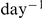	0.0765 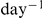	0.0861 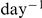
20000 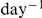	0.0088 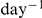	0.0185 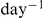

We calculate the activation rates 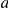 for an initial mean viral load of 35 c/mL, given the virion production rate 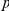. The replication rate 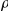 is then chosen so that the half-life of the latent reservoir is 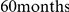.

As noted above, we must perform calculations over the entire blood volume, which we take to be 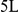. When presenting results below we report viral loads in copies per mL, as this is the standard measurement, but they are always obtained by re-scaling the axes for results over the entire blood volume.

### Initial distributions

In order to correctly simulate viral blips and latent reservoir extinction in patients with established treated infection, we should carefully choose the initial joint distribution 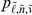 so that it is close to the (moving) equilibrium of the ongoing dynamics. Otherwise, transient effects will pollute our results. In the mean, the dynamics of the latent reservoir are very slow compared to those of the productively infected cells or virions. We therefore focus on getting the initial latent reservoir distribution correct since errors in the other two compartments will resolve themselves quickly. Indeed, for a constant latent reservoir size, and our parameters, the distributions on 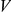 and 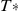 converge to stationary distributions in less than a month (results not shown).

**Figure 2:**
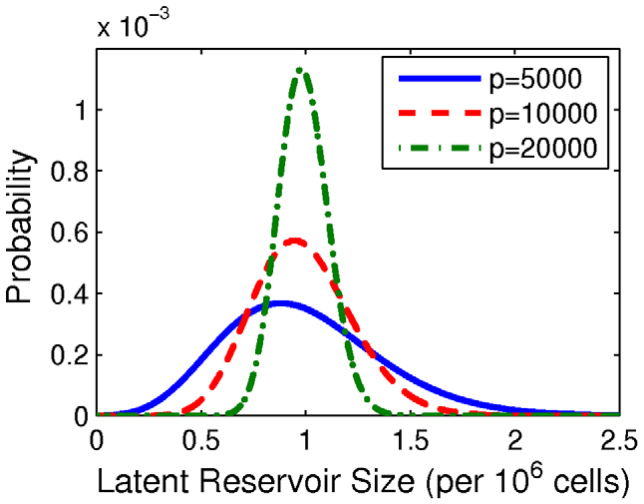
Initial probability distribution on latent reservoir size. We take an initial mean viral load of 25 c/mL and parameters given in Tables 1 and 2. Production rates 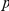 have units 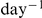. Initial distributions assuming initial mean viral load of 35 c/mL (Table 3) are qualitatively similar (not shown).

In order to calculate a reasonable initial latent reservoir distribution we isolate its dynamics and consider the marginal probability distribution only, as in equation (3). We choose the marginal latent reservoir probability distribution at time 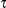 such that the variance is maximized. We reason that transient dynamics on the latent reservoir are dominated by the spreading of the distribution about the decaying mean, and that at maximum variance the probabilities are sufficiently spread for our purposes. For birth-and-death processes maximum variance occurs at the half life 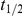. Therefore, in order to create the initial distribution on the latent reservoir 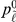, we solve (3) out to 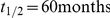, starting with 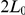 latently infected cells, where 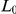 is the desired mean latent reservoir size. The resulting distributions for different parameter sets are shown in Figure 2. Notice that results based on a virus production rate 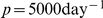 have larger standard deviation. This is because lower production rates are associated with higher activation rates 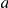 (cf. Tables 2 and 3). The higher activation rate speeds the dynamics of the latent reservoir, increasing the spread of its probability distribution function. Finally, we combine the computed initial latent cell distribution with single initial numbers of productively infected cells and virus, to obtain the whole initial joint probability distribution:
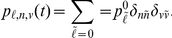



## Results

### Latent reservoir extinction

The reservoir of latently infected cells is considered a major obstacle to clearing HIV infection [19]. Within our model, when the reservoir goes extinct, viral load quickly goes to zero, since ongoing viral replication is too small to sustain the virus population. We are therefore interested in examining the reservoir lifetime after the onset of ART. To do this, we extend our approach to find the probability of reservoir extinction over time. We examine the reservoir lifetime using baseline parameters (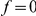), and then allowing for the possibility of latent reservoir replenishment (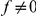). Furthermore, since anti-retroviral treatments have improved substantially over the last 15 years, we also examine how the reservoir lifetime behaves as drug efficacy improves (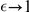).

#### Latent reservoir extinction in the absence of re-seeding

**Figure 3:**
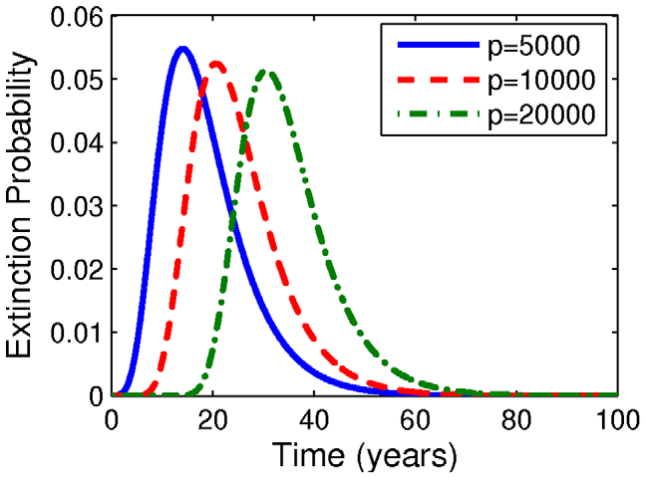
Latent reservoir extinction probability over time. Parameters given in Tables 1 and 2. Production rates 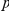 have units 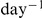.

To begin, we use the baseline parameters (Tables 1 and 2). Since 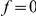, we can use an analytic expression (4). Probability distribution functions for latent reservoir extinction are shown in Figure 3 for each of 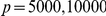 and 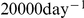. Note that although the production rate 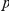 is not explicitly included in (4), the choice of 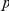 affects the activation and replication rates 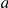 and 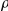 (see Table 2 and the Methods). Figure 3 shows that the resulting distributions are asymmetric and leaning towards longer lifetimes, which is a characteristic of subcritical birth-and-death processes. We also observe that as the production rate 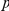 increases (so the activation rate 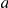 decreases), the distributions shift to the right, with increasing mean and variance. Exact means and variances can be calculating by integrating (4) over time: for 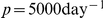 our model predicts a mean reservoir lifetime of 18.7 years with a standard deviation of 8.9 years; for 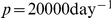, it predicts a mean reservoir lifetime of 34.8 years with a standard deviation of 9.2 years.

At first these results seem surprising: since the reservoir half-life and size are identical in all three calculations, how can the time-to-extinction distributions differ so dramatically? Usually, in a deterministic framework, one would calculate the mean time to extinction assuming exponential decay with extinction when the mean falls below a small threshold. Taking this threshold to be a single cell, it is easy to calculate 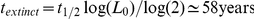. However, the addition of activation and replication events complicates the picture and significantly changes the dynamics once the reservoir gets small. More specifically, the time to extinction decreases as the activation rate 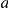 increases, and 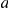 is inversely correlated with production rate 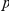 in our model.

However, beyond model- and parameter-specific results, we find an interesting insight: if we wish to predict the timescale for latent reservoir extinction, we must refine our understanding of latent reservoir dynamics beyond half-life estimates to include accurate estimates of birth, death and division, across the various populations that make up the latent reservoir.

#### Latent reservoir extinction with latent cell re-seeding

So far we have assumed that the fraction 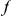 of new infections resulting in latency is zero. Under that assumption, the decay of latent cells is independent of the rest of the model, and in particular, of drug efficacy. However, intensification of drug treatment has been reported to speed latent reservoir decay [51]. To examine this effect, we now consider non-zero 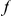 so that latent reservoir replenishment is now in part through infection of new cells (Figure 1). As discussed in the Methods, the overall decay rate of the latent reservoir is kept the same via small corrections (less than two percent) in the replication rate 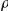.

**Figure 4:**
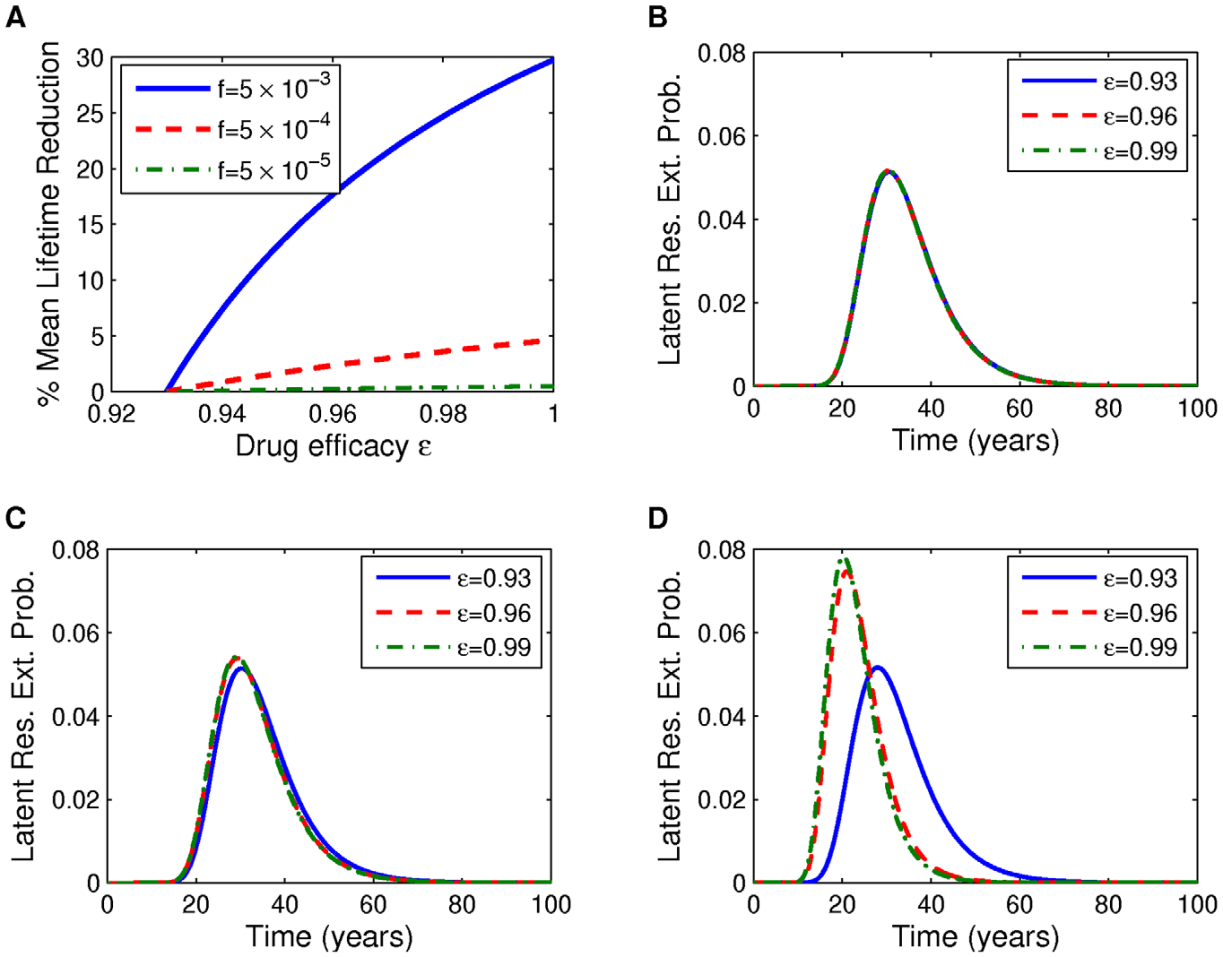
Reductions in latent reservoir lifetime with improving drug efficacy. (A) Percent mean reduction in latent reservoir lifetime with improving drug efficacy 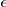. (B–D) Corresponding latent reservoir extinction distributions with improving drug efficacy for fraction 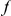 of newly infected cells becoming latently infected (B) 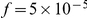, (C) 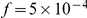, (D) 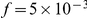. Parameters: Tables 1 and 2 with 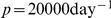.

With improving drug efficacy (as 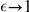) we predict that the mean latent reservoir lifetime decreases, as shown in Figure 4A for the parameters associated with 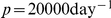. Results for other values of 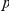 are very similar (not shown). The decrease is less than 1% for 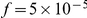 but more dramatic for 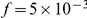, at almost 25%. This reduction is as expected from the model: 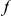 controls the contribution of newly infected cells back to the latent reservoir. But as the drug efficacy is improved, reservoir replenishment is decreased, and the latent reservoir goes extinct more quickly. When 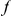 is larger, the contribution of newly infected cells to latent reservoir replenishment is larger. Therefore cutting off this supply results in a more significant reduction in reservoir lifetime.

Furthermore, as the drug efficacy increases (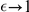), the latent reservoir extinction time distributions narrow, so that the variance decreases and the asymmetric tail shrinks. This effect is illustrated for 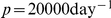 in Figure 4B–D (results for other values of 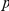 are qualitatively similar, not shown). This is not unexpected: with improving drug efficacy, reservoir replenishment/birth is diminished, which is the source of the asymmetric tail of the distribution. Assuming our model is correct, this is a moderately encouraging result: with improving drug efficacy the range of possible lifetimes is reduced. Nonetheless, in our model we still find latent cell clearance only after decades of drug treatment.

#### Transient loss of free virus

**Figure 5:**
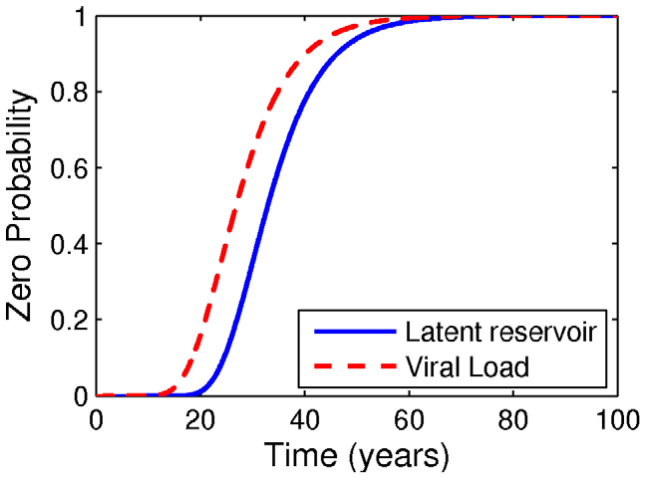
Transient and permanent viral extinction. We plot the probabilities that the viral load is zero (transient viral extinction) and that the latent reservoir is zero (permanent viral extinction) as a function of time. Parameters: Tables 1 and 2, with 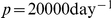.

As described above, the timescale of true viral clearance in our model is set by the decline of the latent reservoir. However, it is possible to transiently achieve 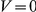 before the latent reservoir has disappeared altogether. In Figure 5 we plot the cumulative distribution function for the first occurrence of this event. Latent reservoir extinction lags transient viral clearance by approximately 6 years, with a mean time of 32 years, for these parameters.

### Viral load distributions in treated patients

**Figure 6:**
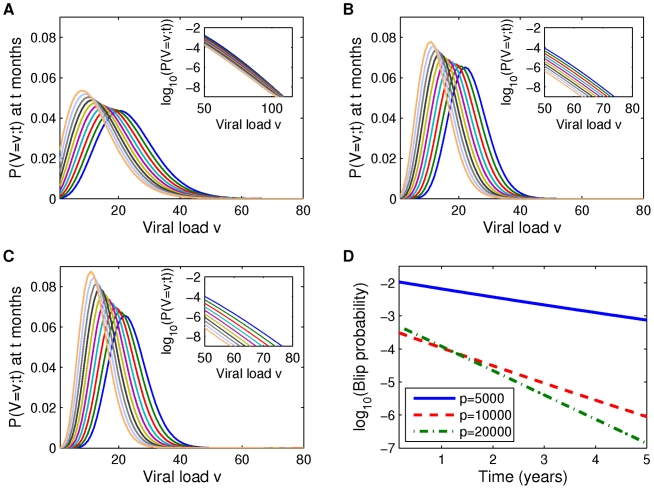
Viral load probability distributions for initial mean viral load of 25 c/ml. (A–C) Distribution functions are plotted at 6 month intervals for parameters given in Tables 1 and 2, and (A) 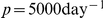, (B) 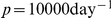, (C) 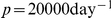. Insets: enlargement of probability distribution curves above the detection level, 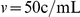; a log scale is used to better distinguish the curves. As time advances the distributions move from right to left. (D) Blip probability plotted against time. The curves in (D) are computed by integrating the probability density functions from (A–C) over viral loads exceeding 50 c/mL.

**Figure 7:**
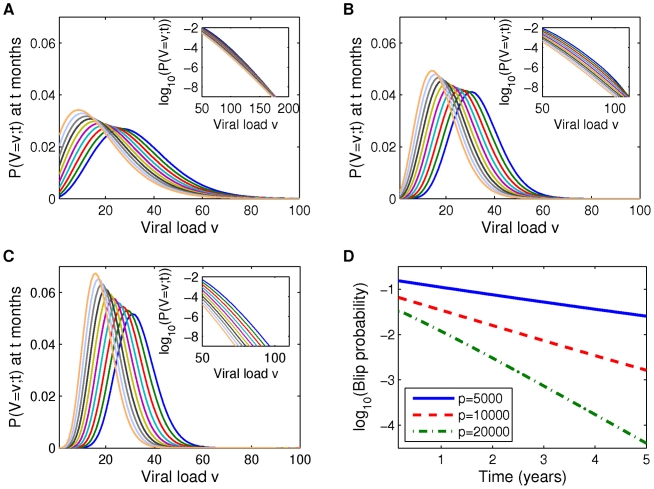
Viral load probability distributions for initial mean viral load of 35 c/ml. (A–C) Distribution functions are plotted at 6 month intervals for parameters given in Tables 1 and 3, and (A) 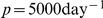, (B) 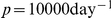, (C) 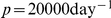. Insets: enlargement of probability distribution curves above the detection level, 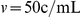; a log scale is used to better distinguish the curves. As time advances the distributions move from right to left. (D) Blip probability plotted against time. The curves in (D) are computed by integrating the probability density functions from (A–C) over viral loads exceeding 50 c/mL.

We now focus on the time evolution of viral load and the likelihood of small-amplitude viral blips. We interpret viral load above the threshold of detection of 50 c/mL as a viral blip. Note that unless otherwise specified, the following calculations and computations assume the fraction 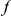 of newly infected cells that become latently infected is 0. In Figures 6 and 7, we plot full viral load distributions over time, assuming initial mean viral loads of 25 c/mL and 35 c/ml. As time advances, the mean viral load decreases as expected in all cases but the viral load distributions widen more significantly when 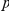 is smaller (e.g. distributions in Figure 6A are widest, those in Figure 6C are narrowest). This is because the lower values of 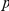 are associated with higher values of 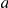 and 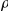, and the resulting dynamics on the latent reservoir cause the latent reservoir size probability distribution (not shown) to be wider. As a consequence the associated viral load distributions are wider, and this also causes higher blip amplitudes. This effect is more clearly understood by examining the insets in Figures 6 and 7, which represents a magnified view of the given probability distribution curves above the blip threshold (50 c/mL), using a log scale to more clearly distinguish the curves. We observe that viral blips occur with very small probability regardless of the production rate 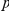. The blip amplitudes vary between parameter sets but remain approximately within the range of blips unassociated with clinical variables shown in [34], i.e. 50–100 c/ml. Over three years, the range of reasonably likely detectable viral loads decays slowly, but small blips remain possible throughout that time (Figure 6).

#### Probability of detectable viremia declines exponentially as a function of time

Figures 6D and 7D show the probability that an individual on ART has a detectable viral load at some given time 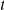, 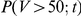. For all parameter sets, the probability of a blip declines exponentially over time. From the equations for mean viral load and latent reservoir size (1) we see that both are decaying exponentially with half life set by 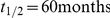. However, blip probabilities decay much more quickly (with half-lives on the range of 6–18 months) than would be predicted from studying the mean behaviour of the system. This underlines the importance of taking a stochastic approach to predicting rare stochastic events.

As expected, in both cases the parameter set associated with the largest latent cell activation rate (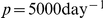) yields the highest blip probability. Predictably, the likelihood of viral blips is substantially higher when the mean viral load is higher, and this is in agreement with a decline in blip detection as drug treatment has improved and reduced setpoint viral load. Our finding that blip probability declines over time disagrees with the report of Di Mascio et al. [31], where a constant rate of blips was observed. We attribute this difference to the fact that many of the blips considered by Di Mascio et al. were of large amplitude rather than the small blips we are examining here. Our basic modeling assumption is that small viral blips under consideration here represent large deviations from a small mean viral load, but that mechanisms for larger viral blips - not fully captured within our model - depend on external factors. Examples of these factors include target cell increase due to immune system activation in response to an unrelated infection, or treatment non-adherence; the frequency of such events is unlikely to change over time, which would result in a constant rate of viral blips over time.

#### Impact of increasing drug efficacy

With our baseline parameters, predicted viral loads are in the range of those measured in [3] but 3–5 times higher than viral loads measured recently [10], [11]. Further, recent clinical observations reveal that blips are now very rare. We can reproduce such observations in our model with improving drug efficacy 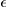. We used a lower drug efficacy of 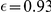 to reflect drug efficacy at a time when viral blips were observed. However as 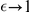, reflecting modern improvements in drug efficacy, we note that both the mean viral load and the variance decrease dramatically. For 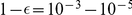, reasonable for current drug regimens [52], we compute (with baseline parameters, Tables 1 and 2) a mean initial viral load of 3–4 c/mL rather than 25 c/mL, and find blip probabilities to be smaller than we can calculate. This implies that, with excellent drug treatment and perfect adherence, any viral blips are almost certainly not due to stochastic reactivation of latent cells.

#### Viral blips driven by secondary infection

Nonetheless, blips continue to be observed in treated patients. One argument for their existence is poor compliance with the drug regimen, effectively reducing the drug efficacy averaged over time. A second possibility is that immune system activation due, for example, to transient secondary infection, can cause episodes of increased viremia. Specifically, during infection, the number of CD4 cells increases to fight infection; more target cells means more infected cells, producing more virus, increasing the viral load to a detectable level [38], [41]. Furthermore, memory T cells make up a part of the latent reservoir and infection may therefore induce an increased activation rate [19].

**Figure 8:**
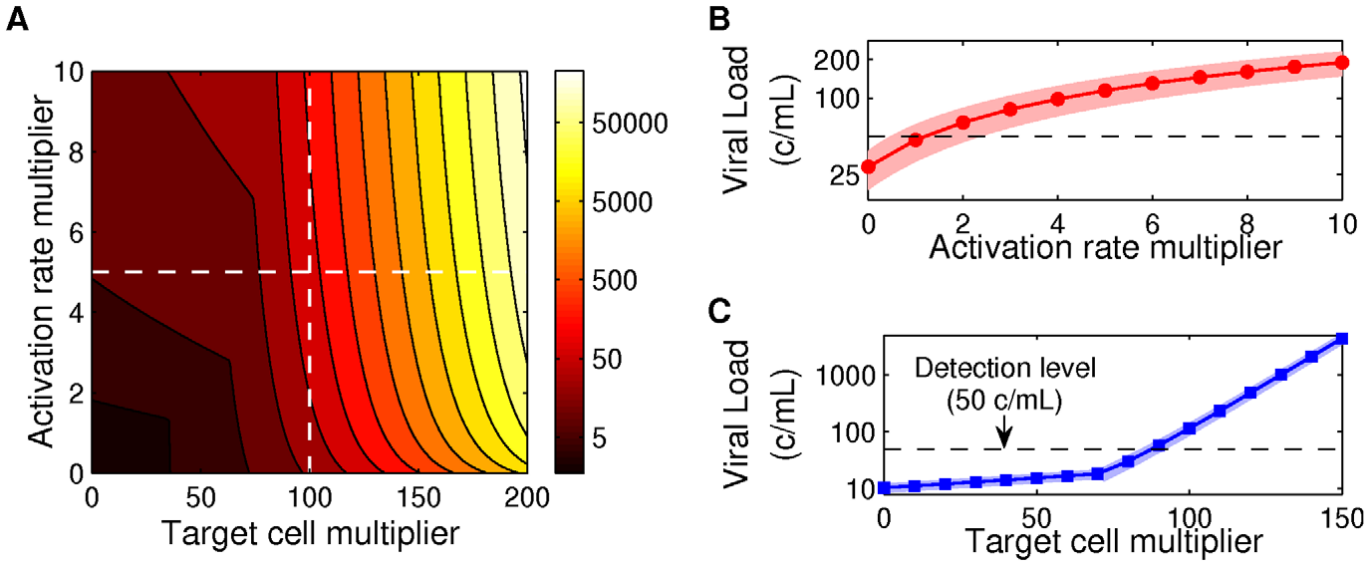
Maximum viral load under immune system activation. (A) Maximum mean viral load for different multiplicative increases in target cell populations 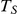 and activation rates 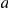. Dashed lines indicate target cell multiplier 100 (vertical) and activation rate multiplier 5 (horizontal). (B) Maximum mean viral load (symbols) 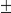 one standard deviation (shaded area) depending on activation rate multiplier, for target cell multiplier 100 (along vertical line in (A)). (C) Maximum mean viral load (symbols) 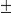 one standard deviation (shaded area) depending on target cell multiplier, for activation rate multiplier 5 (along horizontal line in (A)). Parameters: Tables 1 and 2 with 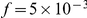, 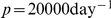 and drug efficacy 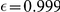.

We experimented with adjusting our model to simulate a transient secondary infection. At initiation, we increase the activation rate 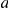 for 3 days and then return to the background value. We also increase the number of target cells 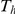 for 7 days, starting after 2 days. These parameter changes must be step-function-like because our method for solving the backwards equation only admits constant rates. Figure 8A shows the mean of maximum viral load for different activation rate and target cell number multipliers, using parameters 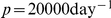, 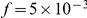 and 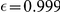. We observe that the increase in target cells has the dominant effect on maximum viral load, although the increased activation rate does provide a boost. Figures 8B and C show the mean of maximum viral load 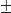 one standard deviation, as a function of activation rate multiplier (for target cell multiplier fixed at 100) and as a function of target cell multiplier (for activation rate multiplier fixed at 5), respectively. The variance is very small. These incomplete results show that for blips associated with immune system activation, the latent reservoir plays only a small role.

### Viral blip durations

In this section we consider the following question: given a blip, defined as a detectable viral load measurement, how long should we expect the viral load to remain above the threshold of detection? This question is of clinical interest, since a repeat measurement following a measurable viral load should be performed after enough time that a second positive result might have clinical significance, such as suggesting drug failure.

Different from the previous sections, all results in this section are computed via 10000 direct simulations of the branching process using the Gillespie algorithm, beginning with an initial “blip” condition. The initial conditions are chosen as follows: we set the latent reservoir size 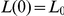 and viral load 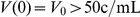. Since dynamics on the viral load 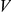 are so much faster than on the productively infected cells 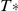 (cf. Table 1), we then use a quasi-steady approximation to set the initial number of productively infected cells 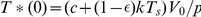.

#### Blip duration dependence on initial viral load measurement

**Figure 9:**
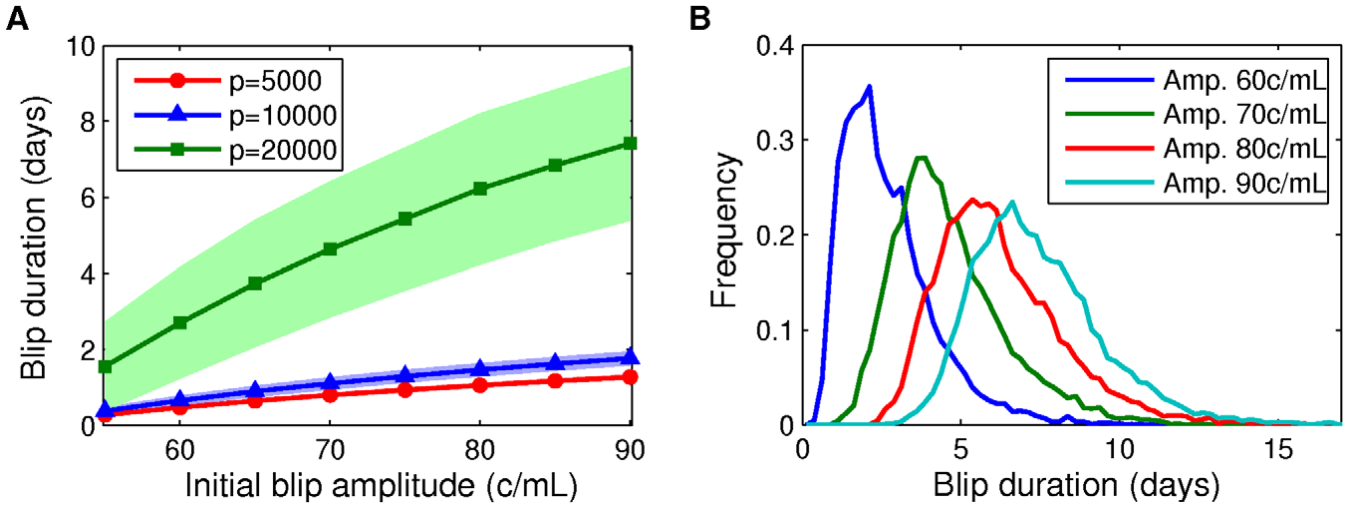
Blip durations depend on initial blip amplitude. (A) Mean blip durations (symbols) 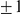 standard deviation (shaded area), computed over 10000 simulations, plotted as a function of the initial viral load measurement (initial blip amplitude). Production rates 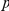 have units 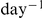. (B) Frequency plots of time distributions of detectable viral load given initial measurements of 60–90 c/mL, computed over 10000 simulations. Parameters: Tables 1 and 2; latent reservoir size 1 per 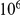 cells; 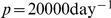 in (B).

**Figure 10:**
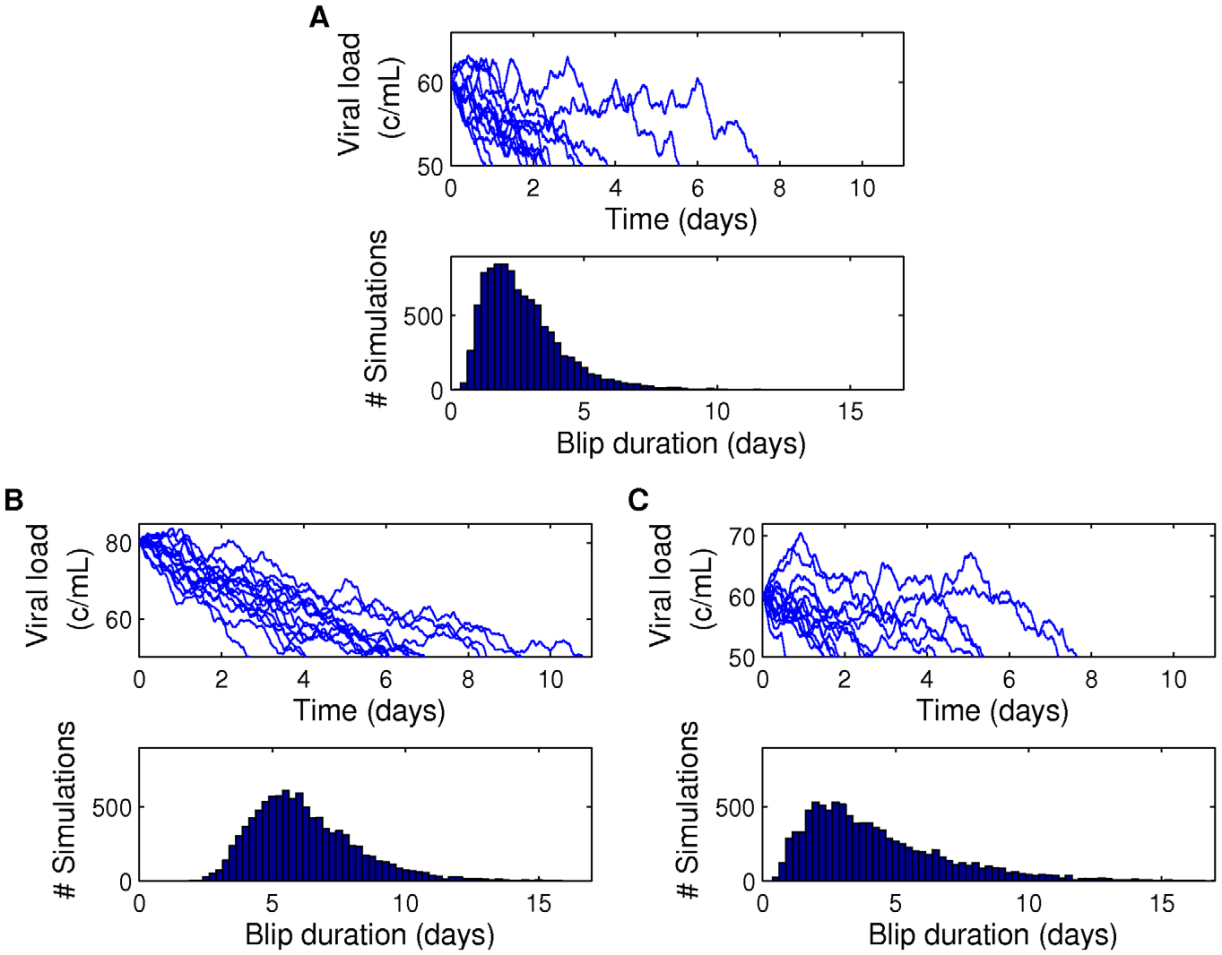
Realizations of Gillespie simulations showing viral load evolution. (A–C) show sample viral load evolutions and the associated histogram of durations until the viral load is below 50, over 10000 simulations, given an initial viral load measurement and latent reservoir size. (A) Initial viral load measurement of 60 c/mL with latent reservoir size 1 per 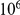 cells; (B) initial viral load measurement of 80 c/mL with latent reservoir size 1 per 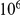 cells; (C) initial viral load measurement of 60 c/mL with latent reservoir size 1.5 per 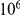 cells. Parameters: Tables 1 and 2, for 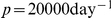.

We begin by calculating blip durations as a function of a detectable viral load measurement at time zero. Figure 9A shows the mean blip duration 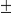
1 standard deviation, over different values of the production rate 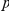, with the latent reservoir size set to be 1 cell per 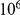. As the initial measured blip amplitude increases, so do the mean and standard deviation of the blip duration. This is because the more productively infected cells there are, the longer it takes for enough of them to die and therefore reduce the viral load. Also, since mean duration is longer for larger blips we also expect a larger standard deviation, since there is more opportunity for variability. The mean duration increases with increasing production rate, since for higher 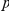 more virions are produced, and have the opportunity to infect healthy cells, before the productively infected cells die. Duration distributions for different initial detectable viral load measurements are shown in Figure 9B for 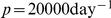. From this figure we see that the standard deviation alone does not fully determine blip duration variability. For smaller blip amplitudes the distribution is more asymmetric, with a relatively larger probability of longer blips (positive skew). As initial blip amplitude increases, the distribution becomes more symmetric. This observation is explained by noting that as the initial blip size is increased, we are moving the initial condition farther and farther from equilibrium. Therefore, the stochastic dynamics are increasingly driven by decay of 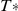 and 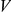 towards the equilibrium, and it becomes increasingly unlikely for the viral load to increase after the initial measurement. Direct observation of viral load evolution in different realizations of master equation simulations support this argument, as shown in Figures 10A and B. For example, after an initial measurement of amplitude 60 c/mL (Figure 10A), the viral load does not decay as rapidly as it does for a measurement of 80 c/mL (Figure 10B).

#### Effect of latent reservoir size on blip duration

**Figure 11:**
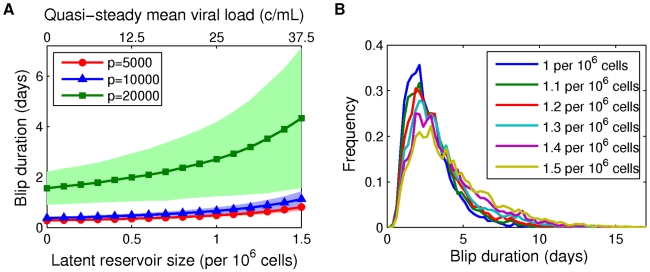
Blip durations depend on initial latent reservoir size. Mean blip durations (symbols) 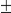
1 standard deviation (shaded area), computed over 10000 simulations, plotted as a function of the initial latent reservoir size. (B) Frequency plots of time distributions of detectable viral load given initial latent reservoir sizes of 1–1.5 cells per 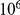. Parameters: Tables 1 and 2; initial viral load measurement of 60 c/mL; 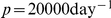 in (B).

We expect that larger reservoir sizes should be associated with longer blips, since a higher reservoir size is associated with a higher quasi-steady mean viral load. Our results confirm this expectation. Figure 11A shows the mean and standard deviation of an amplitude-60 c/mL blip increasing with the reservoir size, for latent reservoir sizes 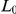 between 0 and 1.5 cells per 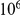. As the reservoir size nears 2 cells per 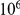 we anticipate that the duration gets very large: at this level, the associated quasi-steady viral load is above the detection threshold of 50 c/mL, and we must wait until the viral load decays naturally to a mean below that threshold. This is extremely unlikely in our model: the probability that the latent reservoir size reaches 2 cells per 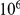 is initially very small 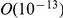 for 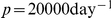 (see Figure 2) and only decreases over time. Notice also in Figure 11A that, as before, larger production rates 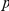 result in blips with longer durations.

We plot duration distributions across different latent reservoir sizes in Figure 11B. Interestingly, although the mean duration increases with reservoir size as shown in Figure 10A, the peak of the distribution stays in the range of 2–3 days. Therefore our modeling suggests that regardless of reservoir size, given a viral load measurement of 60 c/mL, the viral load in most patients should drop below detection level after 2–3 days. In contrast to the results with increasing initial blip measurement, we observe that the asymmetry in blip duration distributions is increasing - the tail is getting heavier with increasing reservoir size. This is because larger reservoir sizes are associated with larger associated mean viral loads (see Figure 11A). For the smaller reservoir sizes, the initial viral load of 60 c/mL is further away from the associated mean and viral decline is therefore quicker. Again this is supported by direct observation of viral load evolution in different simulations (Figures 10A and C).

## Discussion

We have presented a simple but fully stochastic model of HIV viral dynamics in individuals on antiretroviral treatment, focusing in particular on the role of the latent reservoir. In our model we included dynamics of only three compartments: the numbers of latently infected cells 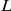, productively infected cells 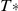, and virions 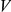. We assumed that all rates correspond to exponentially-distributed transition probabilities and that therefore dynamics could be described by a continuous-time, multi-type branching process. We then derived equations for the probability generating function and using novel numerical techniques we computed the probability distributions on viral load over time, recovering features that are hard to study with approaches based on differential equations or direct simulation.

Our model reproduces interesting features of successfully treated infection, namely a usually low, undetectable viral load [3] and brief periods of low-amplitude detectable viral load, unassociated with clinical or demographic parameters, as discussed in [34]. This shows that the hypothesis that random activation of latent cells plays a major role in residual viremia on treatment, as has has been suggested by clinical evidence (e.g. [9]), is reasonable and is compatible with reasonable parameter estimates. We were also able to use our model to look at the slow decline of the latent reservoir itself.

### Latent reservoir extinction

Clinical results on latent reservoir decay (e.g. [21]) make predictions on latent reservoir lifetimes that are based on purely exponential decay. Our model results showed that, for the same mean decay rate, the time distribution - and the mean time to extinction - is sensitive to dynamics on the latent reservoir. Further, assuming some reservoir replenishment due to latency in newly infected cells, our model predicted only limited lifetime reduction associated with improving drug efficacy. Eradication of the latent reservoir is considered a major hurdle in eradicating HIV infection [19], [23], and these results demonstrate the importance of understanding the underlying dynamics on the latent reservoir: the reservoir half-life is only a small part of the equation.

From a clinical point of view our results on the latent reservoir lifetime are quite depressing - in our model we essentially study perfectly drug-adherent patients, and even with perfect drugs, decades of drug treatment are needed to clear the latent cell reservoir. Our model predicts that drug treatments that increase the activation rate of latently infected cells should reduce the lifetime of the reservoir. This approach has been tried several times but so far without real success (reviews in [1], [53]). A candidate drug would need to work on the whole heterogeneous population of cells seeded during initial infection, and in particular on the longest-lived subpopulation. We would predict that early treatment with such a drug, along with aggressive ART, would be most likely to reduce the size of the latent reservoir. This is in line with current research in treating HIV infection earlier, to enhance survival on an individual level [54] and limit transmission on the population level [55]. Of concern with earlier treatment is the possibility of emergent drug resistance (DR). In future work we plan to expand our stochastic model to examine the likelihood of different mechanisms of acquired DR in patients on treatment, such as mutation during ongoing viral replication and activation of a cell latently infected with a DR strain [56].

### Viral blip frequency over time

We also examined the evolution of viral load over time, finding that as time progresses, the viral load distributions become more asymmetric, with a long tail towards higher viral loads. This can be explained by viewing our model as an extended subcritical birth-and-death process. Such processes produce asymmetric distributions (see Figures 6 and 7). The asymmetry is more pronounced for smaller production rates (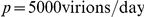 vs 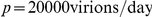), associated with larger activation rates, and for larger initial viral loads (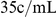 vs 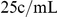). When examining blip probability we found that our model predicts that these probabilities decay exponentially over time. This decay is more dramatic for larger production rates, associated with smaller activation (‘birth’) rates, and smaller initial mean viral loads. In only one case is the decay so slow (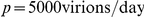 with mean initial viral load 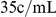) that our model predictions are broadly consistent with previous observations that blip probabilities don't decay over time [45]. We also observed that blip probabilities show great sensitivity to model parameters, varying by orders of magnitude. Given sufficient high-quality data on blips (which does not currently exist), these would be the ideal results to compare with data for the purpose of parameter fitting, in order to gain some insight into latent reservoir dynamics - into the activation rate 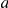, for example.

With improving drug efficacy (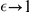) our model predicts a significant decrease in baseline viral load, down to 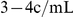, in accordance with recent viral load observations [10], [11]. From this low baseline, we found blip probabilities smaller than we can calculate. However, we found that by increasing the activation rate (roughly simulating immune system activation due to secondary infection) viral loads exceeding the threshold of detection were attainable. Therefore, our model supports the hypothesis that, for patients adhering to modern ART, viral blips signal an underlying secondary condition.

### Duration of viral blips

We examined the duration of viral blips through direct (Gillespie) simulation of the model. We sought to answer the question “Given a patient measurement of X c/mL, how long can we expect the viral load to remain detectable?” We found, unsurprisingly, that blips of larger initial amplitude have longer mean duration and larger standard deviation in duration. Perhaps more interestingly, we found very strong dependence on the production rate 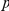. Given an initial blip of amplitude 90 c/mL, doubling the production rate from 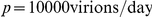 to 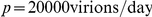, and changing other parameters accordingly, more than triples the predicted mean blip duration (for parameters as in Figure 9). We also considered blip duration as a function of the latent reservoir size, anticipating longer durations for larger reservoir sizes, since associated with these is a higher quasi-steady mean viral load. Our expectations were confirmed by simulation results (see Figure 11). The sensitivity to production rate 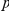 and associated parameters was also recovered.

Repeat-blip measurements in patients are, predictably, rather rare, since blips are already quite unusual events. Across all the parameter sets we examined, we found that detectable viremia should be expected to vanish within 8–10 days at most. This result is in general agreement with previous reports [34], [45] and indicates that repeat low-level detectable viremia within 8–10 days could be due to a statistical fluctuation rather than drug resistance or other pathology.

### Stochastic modeling of viral infection

Over the last 15 years, enormous numbers of differential-equation models have been generated to study different aspects of various viral infections. We believe that stochastic models of the kind described here have an important role to play in certain situations where viral or cell populations are small enough that random effects still play a role. The obvious settings are during the first few days of any new infection (see also [57] and, very recently, [58]), during drug treatment of a chronic infection, and during the extinction phase of an acute infection. One issue with stochastic modeling of rare events (such as viral blips in our model) is that simulation-driven studies can require enormous numbers of simulations to reliably sample the rare events. The method we describe here is an alternative to simulation (or methods to capture rare-events) and provides a direct and relatively straightforward way to calculate probability distribution functions. We hope to adapt this method to other situations in viral dynamics in future work.

## Supporting Information

Figure S1Comparison between our probability distribution function calculations and direct numerical simulations using the Gillespie algorithm. Distributions over the number of productively infected cells are plotted at 1 year, starting with 1 per 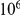 latently infected cells only, for parameters given in Tables 1 and 2. (A–C) Frequencies over 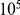 stochastic simulations are compared to probability distributions derived using our method, for (A) 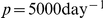, (B) 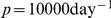 (C) 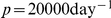. (D) Enlargement of tail in (C), using a log scale for clarity, with frequencies over 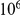 Gillespie simulations. Notice that direct calculation of the probability distribution is clearly preferable to simulation when rare events are studied.(TIFF)
