# Supplementary figures and images for: A Stochastic Model of Latently Infected Cell Reactivation and Viral Blip Generation in Treated HIV Patients
Source: PLoS Comput Biol. 2011 Apr 28;7(4):e1002033. doi: 10.1371/journal.pcbi.1002033 (PMC3084212; doi:10.1371/journal.pcbi.1002033)

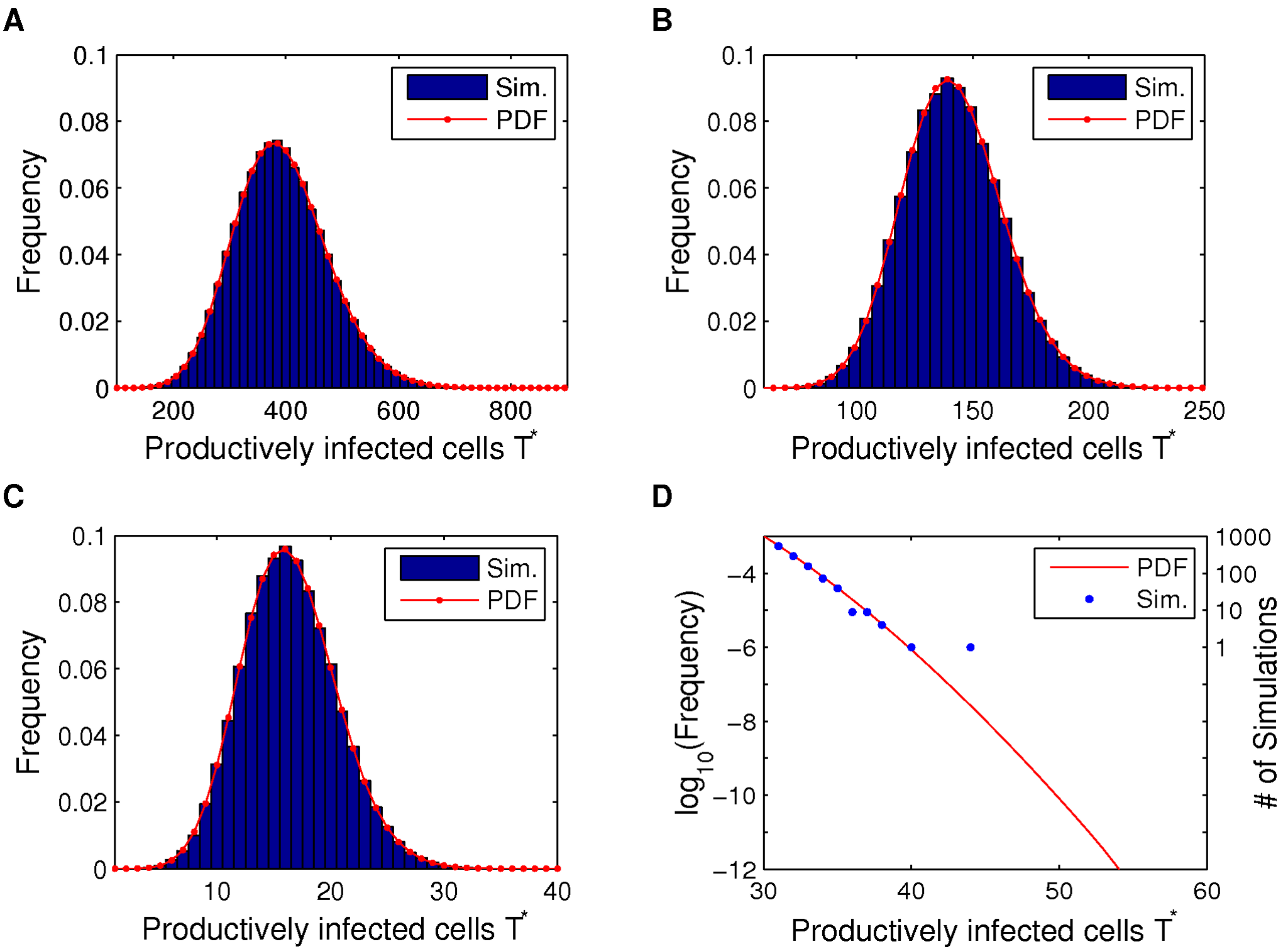

Supplement: Figure S1 — Comparison between our probability distribution function calculations and direct numerical simulations using the Gillespie algorithm. Distributions over the number of productively infected cells are plotted at 1 year, starting with 1 per latently infected cells only, for parameters given in Tables 1 and 2. (A–C) Frequencies over stochastic simulations are compared to probability distributions derived using our method, for (A) , (B) (C) . (D) Enlargement of tail in (C), using a log scale for clarity, with frequencies over Gillespie simulations. Notice that direct calculation of the probability distribution is clearly preferable to simulation when rare events are studied. (TIFF) [file pcbi.1002033.s001.tiff]
